# Patterns and stakeholder-perceived drivers of Caesarean section practices: Evidence from a multicenter study in a lower-middle-income country

**DOI:** 10.1371/journal.pgph.0006274

**Published:** 2026-05-14

**Authors:** Maria Atif, Waqas Ahmed Farooqui, Shazia Jabbar, Mubasshir Saleem, Jahan Ara Hasan

**Affiliations:** 1 College of Public Health, Ziauddin University, Karachi, Pakistan; 2 School of Public Health, Dow University of Health Sciences, Karachi, Pakistan; 3 Department of Obstetrics, and Gynecology, Dow University of Health Sciences, Karachi, Pakistan; 4 Department of General Medicine, University of Alberta, Edmonton, Alberta, Canada; ICDDR: International Centre for Diarrhoeal Disease Research Bangladesh, BANGLADESH

## Abstract

This mixed-method study aimed to generate local evidence on the patterns of cases of C-Sections and to qualitatively explore perceptions of stakeholders regarding the drivers of current C-Sections practices and WHO-recommended, evidence-based interventions for reducing unnecessary C-Sections in Pakistan. The study population included 605 women who underwent C-Sections in either public, private or semi-private healthcare facilities. A prior history of C-Section was the most common reason for subsequent C-Sections, with Group 5 being (multiparous women with a previous C-section, carrying a singleton fetus in cephalic presentation at term) the greatest contributor to the overall C-Section rate. Qualitative data revealed that a majority of women do not prefer C-Sections mainly due to the prolonged recovery time associated with it; however, pain associated with vaginal birth may be a compelling factor for women to opt for C-Sections. Improved birth preparedness and counselling on pain management may be appropriate non-clinical interventions to reduce unnecessary C-Sections in Pakistan. There is an expressed need to implement WHO-recommended guidelines on non-clinical interventions to reduce unnecessary C-Sections in Pakistan.

## Introduction

Caesarean Sections (C-Sections) are considered necessary emergency procedures to prevent perinatal morbidity and mortality, and C-Section rate at the population level is a vital measure of quality and level of access to obstetric services, enabling policy makers in evaluating maternal and child health and utilization of emergency obstetrics care and resources [1,2]. Since 1985, the acceptable rate of C-Sections suggested by the WHO is 10–15%; however, these rates have far surpassed in some countries, and there is limited data to support a significant improvement in maternal mortality with C-Section rates increasing beyond 10% [2,3]. The current WHO guidelines suggest that there is no recommended “ideal” rate, but that caesarean sections should be performed when medically indicated [2].

An analysis of global trends and frequency of C-Sections reported that 29·7 million of 140·6 million live births were estimated to be by C-Sections in 2015, and within individual countries, C-Section was found to be highest among wealthier women and in private facilities [4]. South Asia has experienced the second-largest absolute increase in C-Sections, jumping from 7.2% in 2000 to 19.2% in 2014 [5]. However, a few European countries including, Finland, Norway, and Iceland have managed to significantly reduce their C-Section rates to around 15% [6].

Literature has identified several factors contributing to increased C-Sections worldwide, with financial incentives, maternal requests, and lack of regulations being the most significant factors [7–10]. Despite its significance as an emergency life-saving obstetric procedure to ensure maternal and neonatal wellbeing, evidence suggests that C-Sections is positively associated with maternal mortality, maternal and neonatal morbidity, and increased short and long-term complications that can sometimes be permanent and are more prevalent in low-resource settings [11,12]. The economic costs of C-Sections are especially concerning for lower-and middle- income countries. A study conducted in Bangladesh estimated that the average medical care expenditure for a C-Section was USD 276 in 2010, compared to USD 45, the cost of vaginal birth [13].

Considering the alarming increase in the rates of C-Sections and the associated adversities, the WHO has recommended using the Robson classification system, which classifies all births into ten mutually exclusive and collectively exhaustive groups based on a set of predefined obstetric parameters [14]. This classification system enables auditing and analysing C-Section rates, and allows the identification of the groups that make significant contributions to the overall C-Section rate, which can enable healthcare providers (HCPs) to analyse the degree of necessity for a C-Section, thus providing an opportunity to avoid all unnecessary C-Sections [1,14]. In 2018, the WHO developed a comprehensive guideline on recommendations based on global evidence on the effectiveness of non-clinical interventions, which can be used to reduce unnecessary C-Sections [15]. A 2019 umbrella review of 101 systematic reviews and ten reports of evidence-based recommendations have also identified several clinical and non-clinical interventions that can effectively reduce the rates of unnecessary C-Sections [12]. Another systematic review of global literature suggested that the interventions to reduce unnecessary C-Sections should be multifaceted and locally tailored to improve the acceptability and applicability of such interventions [16].

In Pakistan, C-Sections have increased consistently from 3.2% in 1990–91–22% in 2017–18, suggesting that Pakistan is following a global trend of having C-Sections for non-medical reasons [17]. A few studies have analysed the reasons for increasing rates of C-Sections in Pakistan, however, their findings have been inconsistent and such studies lack recommendations to reduce the rates of C-Sections in Pakistan [17,18]. Moreover, such sporadic studies lack the generalisability required to represent the true extent of practical utilisation of the WHO 10-group classification in Pakistan. This study was, therefore, designed to generate local evidence on the the patterns of C- Section deliveries, stakeholder-perceived drivers of cases of C-Sections and to qualitatively explore perceptions of stakeholders regarding the WHO-recommended, evidence-based interventions for reducing unnecessary C-Sections in Pakistan.

## Materials and methods‌‌

### Study design and setting

This multi-center, mixed-methods research study was conducted in Karachi, Pakistan. Data were collected from 1^st^ June 2024–31^st^ October 2024, from a government, a semi-private, and a private healthcare setting (HCS). Semi-private HCS is a hybrid model, combining features of public and private HCSs in terms of cost and quality. A mixed-methods approach was adopted to assess both quantitative determinants and qualitative stakeholder perspectives on caesarean section practices. The study population included 605 women who underwent C-Section at the selected HCS.

### Ethics statement

The Institutional Review Board of Dow University of Health Sciences granted the ethical approval (IRB-2848/DUHS/Approval/2023/11), and this study was conducted in line with the Declaration of Helsinki, with written informed consent obtained from the participants prior to the data collection.

We used convenience sampling and a descriptive cross-sectional methodology to assess current C-Section practices against the WHO 10-group classification system. All women aged ≥18 years who had given birth through C-Section at the selected HCS, during the study data collection period were eligible to participate in this study, while women who had laparotomy done for uterine rupture were excluded. The sample size of 533 subjects was calculated using PASS version 15 software, based on the test for one sample proportion with a 95% confidence interval, 5% margin of error, and the most common three 10-Group Classification System (Group-10) proportion [18] in pregnant women (77%).

In parallel, we purposively selected key stakeholders—including postpartum women who underwent C-Section, obstetricians, and hospital administrators at the selected HCS for qualitative interviews to explore perspectives on C-Section practices. The study also explored stakeholders’ perspectives towards the implementation of the WHO-recommended interventions for reducing unnecessary C-Section and their adaptability in the Pakistani context. A sample size calculation was not applied for the qualitative component, and interviews continued until data saturation was reached. The qualitative interviews with the consenting postpartum women were conducted at the doctors’ examination rooms, second post-operative day to ensure adequate recovery of postpartum women before they were discharged from the hospitals. The IDIs with obstetricians and hospital administrators were conducted at their respective offices.

### Data collection and analysis

The data collection commenced with the distribution of the Participants’ Information Statements (introducing the study to the eligible participants) and consent forms among 618 eligible post-partum women (to account for any incomplete data, we approached 618 participants). However, 605 post-partum women provided their written consent and were included in the study.

A structured questionnaire was used to gather data (S1 File). Demographic and socio-cultural Variables (e.g., age, religion, preference for sex of child, employment status, and household income) were obtained directly from participating women, while clinical information (e.g., pregnancy complications, delivery outcomes, etc.) were extracted from medical records. Gestational age ≥ 37 completed weeks was considered a term pregnancy [19]. The questionnaire also included the 10-Groups of the WHO-recommended classification system to categorize all C-Sections. The quantitative data were entered in Microsoft Excel and analysed on IBM SPSS Statistics version 27 (IBM Inc., Armonk, NY, USA). Normality of continuous variables was assessed, and the median and interquartile range were calculated for age and income. Categorical variables, including demographic, clinical, and mode of birth characteristics, were presented as frequencies and percentages. Associations between categorical variables were assessed using the Chi-square or Fisher’s exact tests, as appropriate. A p-value of ≤0.05 was considered significant.

For the qualitative data collection, we conducted in-depth interviews (IDIs) with the stakeholders to understand their perceptions regarding the current C-Section practices in Pakistan and the utility of the WHO-recommended evidence-based interventions for reducing unnecessary C-Sections and the adaptability of these interventions in the Pakistani socio-cultural context. Following a semi-structured guideline for the IDIs, we utilized a thematic analysis technique and an interpretive approach to comprehensively understand prevalent perceptions on the topic. These interviews were transcribed verbatim, analysed in Urdu, and results were reported in English. Two researchers independently read the transcribed interviews and analysed and reported the findings of the qualitative interviews.

### Results of the quantitative component of the study

A total of 605 women (205 from the public sector, 200 from the private, and 200 from the semi-private sector) who underwent C-section were included in the study. The mean age of participants was 27.4 years (SD = 5.6, range: 18–45 years). The majority of women were multiparous (72.4%, n = 438). The demographic characteristics of the sample are described in Table 1. Data are attached as Supplementary files (S1 Data and S1 Text)

**Table 1:** Descriptive statistics of demographic characteristics according to the place of birthing.

Demographic characteristics	Private N = 200 (%)	Public N = 205 (%)	Semi-Private N = 200 (%)
**Age (years)**			
Median (IQR)	27 (23–30)	27 (24–30)	26 (25–30)
Min – Max	19 - 40	18 - 40	19 - 45
**Religion**			
Islam	197 (98.5)	201 (98)	198 (99)
Christianity	2 (1)	1 (0.5)	2 (1)
Hinduism	1 (0.5)	3 (1.5)	0 (0)
Sikhism	0 (0)	0 (0)	0 (0)
Other	0 (0)	0 (0)	0 (0)
**Education**			
None	0 (0)	62 (30.2)	40 (20)
Primary	12 (6)	69 (33.7)	26 (13)
Secondary	57 (28.5)	67 (32.7)	58 (29)
Graduate	122 (61)	7 (3.4)	63 (31.5)
Postgraduate	9 (4.5)	0 (0)	13 (6.5)
Current Employment*			
Employed/Self Employed	38 (19.1)	23 (11.2)	56 (28)
Unemployed	161 (80.9)	182 (88.8)	144 (72)
**Income’000 (PKR)**			
Median (IQR)	50 (40-70)	25 (20212223242526272829–30)	40 (25-50)
Min – Max	20 – 400	6 – 70	1 – 200

*An individual entry missing, IQR: interquartile range

Among women who gave birth in a public-sector hospital, 29% had three or more children. For 63.4% of women who gave birth at the public-sector hospital, the recent pregnancy was unplanned, while for private and semi-private facilities, 46.5% and 57.5% of women respectively reported an unplanned pregnancy. A majority of the women reported having four or more antenatal care visits during their last pregnancy (Table 2).

**Table 2:** Pregnancy-related characteristics of participants.

Participants Characteristics	Private N = 200 (%)	Public N = 205 (%)	Semi- Private N = 200 (%)	P-value	Test statisic and df***
**History of abortion**					
No	143 (71.5)	143 (70.1)	123 (61.5)		
Yes	57 (28.5)	61 (29.9)	77 (38.5)	0.068^C**^	5.34(df = 2)
**Previous C-Section**					
No	74 (37)	138 (67.3)	78 (39)		
Yes	126 (63)	67 (32.7)	122 (61)	<0.001^C**^	46.83(df = 2)
**Pregnancy Planned**					
No	93 (46.5)	130 (63.4	115 (57.5)		
Yes	107 (53.5)	75 (36.6)	85 (42.5)	<0.001^C**^	12.07(df = 2)
Preference of sex of child in recent pregnancy*					
Girl	20 (10)	32 (15.7)	12 (6)		
Boy	75 (37.5)	71 (34.8)	14 (7)	<0.001^C**^	79.91(df = 4)
No preference	105 (52.5)	101 (49.5)	174 (87)		
Had concerns about recent pregnancy*					
No	138 (69)	134 (65.7)	175 (87.5)		
Yes	62 (31)	70 (34.3)	25 (12.5)	<0.001^C**^	28.87(df = 2)
Had complications during recent pregnancy*					
No	69 (34.8)	96 (47.1)	8 (4)		
Yes	131 (65.2)	109 (52.9)	192 (96)	<0.001^C**^	96.05(df = 2)
**Antenatal care visits**					
None	1 (0.5)	6 (3)	0 (0)		
1-3	13 (6.5)	36 (17.7)	7 (3.5)	<0.001^F**^	36.91(df = 4)
4 or more	186 (93)	161 (79.3)	193 (96.5)		

* Index pregnancy (the most recent pregnancy that resulted in the caesarean section under study).

** ^F^Fisher exact test, ^C^Chi-square test.

*** Degree of freedom.

The proportion of C-Sections conducted before the onset of labour was highest (82%) among women attending the private HCS. Emergency C-Sections were performed mostly in the public-sector hospital (81%), and the most frequently mentioned reasons for performing the last C-Sections among all the groups were having a previous C-Section (44%), followed by fetal distress(30%), and maternal request (13%). (Table 3).

**Table 3:** Description of obstetrics characteristics at birth.

Obstetrics characteristics	Private N = 200 (%)	Public N = 205 (%)	Semi- Private N = 200 (%)	P-value	Test statistic and df**
**Gestational age (weeks)**					
<37 weeks	40 (20)	61 (29.8)	69 (34.5)		
≥37 weeks	159 (79.5)	142 (69.3)	131 (65.5)	0.005^F*^	12.23(df = 4)
Don’t Know	1 (0.5)	2 (1)	0 (0)		
**Onset of Labor**					
Spontaneous	15 (7.5)	129 (62.9)	82 (41)		
Induced	21 (10.5)	11 (5.4)	54 (27)	<0.001^c*^	178.26(df = 4)
CS before onset of labor	164 (82)	65 (31.7)	64 (32)		
**Fetal presentation/ lie**					
Cephalic	157 (78.5)	183 (89.3)	188 (94)		
Breech	38 (19)	18 (8.8)	8 (4)	<0.001^F*^	28.52(df = 4)
Transverse/Oblique	5 (2.5)	4 (2)	4 (2)		
**Number of Fetus**					
Single	194 (97)	200 (97.6)	197 (98.5)	0.631^F*^	1.01(df = 2)
Multiple	6 (3)	5 (2.4)	3 (1.5)		
**Mode of C-Section**					
Emergency	123 (61.5)	165 (80.5)	92 (46)	<0.001^C*^	54.25(df = 2)
Scheduled	77 (38.5)	40 (19.5)	108 (54)		
**Fetal Outcome**					
Live birth	197 (98.5)	201 (98)	199 (99.5)	0.548^F*^	1.07(df = 2)
Still birth	3 (1.5)	4 (2)	1 (0.5)		
**Birth weight**					
1,000--2,499	75 (37.5)	37 (18)	108 (54.8)	<0.001^F^*	77.46(df = 4)
2,500--3,999	123 (61.5)	167 (81.5)	80 (40.6)		
≥4,000	2 (1)	1 (0.5)	0 (0)		

* ^F^Fisher exact test, ^C^Chi-square test.

** Degree of freedom.

Most women who gave birth at the public-sector hospital mentioned having a previous C-Section(65%), which led to their recent C-Section. More than 30% of the women who gave birth at a private HCS mentioned fetal distress as a reason for performing C-Section. (Table 4).

**Table 4:** Most frequently recorded reasons for performing the last C-Section in different healthcare sectors.

Reasons*	Private N = 200 (%)	Public N = 205 (%)	Semi-Private N = 200 (%)
Previous C-Section	40 (20)	133 (64.9)	93 (46.5)
Fetal distress	61 (30.5)	44 (21.5)	22 (11)
Maternal requests	56 (28)	0 (0)	6 (3)
Hypertensive disorders of pregnancy	6 (3)	25 (12.2)	16 (8)
Abnormal lie	9 (4.5)	17 (8.3)	11 (5.5)

* only top five reasons mentioned in the medical records are reported.

Binary logistic regression analysis was performed to identify independent predictors of emergency versus elective cesarean section. The final model included 12 variables based on clinical relevance. The model demonstrated good fit (Hosmer-Lemeshow χ² = 8.24, p = 0.41) and acceptable discrimination (AUC = 0.78, 95% CI: 0.74-0.82). Data showed that previous C-section increased odds of emergency CS by 3-fold (AOR = 3.06, 95% CI: 2.08-4.51). Non-cephalic presentation was the strongest predictor for emergency C-section (breech (AOR = 6.62) and transverse/other (AOR = 10.07)), while antenatal complications doubled the odds of emergency C-section (AOR = 2.44). Higher education was found to be a protective factor against emergency C-section (AOR = 0.60, 95% CI: 0.38-0.95), while induced labor increased emergency C-section risk 4-fold (AOR = 4.18) (Table 5).

**Table 5:** Multivariable logistic regression analysis for emergency C-Section.

Variable	β Coefficient	Adjusted OR	95% CI	p-value
**Age (years)**	0.03	1.03	0.99-1.07	0.124
**Education (ref: Primary)**				
Secondary	-0.28	0.76	0.48-1.19	0.225
Higher	-0.51	0.60	0.38-0.95	0.028
**Income**	-0.02	0.98	0.96-1.01	0.187
**Previous C-Section**	1.12	3.06	2.08-4.51	<0.001
**Total children**	-0.15	0.86	0.74-1.01	0.062
**Antenatal complications**	0.89	2.44	1.62-3.67	<0.001
**Fetal Presentation (ref: Cephalic)**				
Breech	1.89	6.62	3.84-11.41	<0.001
Transverse/other	2.31	10.07	4.56-22.24	<0.001
**Onset of labour (ref: Spontaneous)**				
Induced	1.43	4.18	2.51-6.96	<0.001
**Multiple fetus**	0.76	2.14	1.12-4.09	0.021
**Planned pregnancy**	-0.42	0.66	0.44-0.98	0.039
**History of abortion**	0.31	1.36	0.91-2.03	0.131
**Concerns about pregnancy**	0.52	1.68	1.08-2.61	0.022
**Constant**	-2.84	0.06		<0.001

Among all the HCS, the most common WHO group classification was 5. The second most common group classification for public and semi-private HCSs was 10, while the second most common classification for private HCS was group 1 (Table 6).

**Table 6:** WHO 10-Group classification.

Group Classification	Private	Public	Semi-private	P-value
	N = 200(%)	N = 205(%)	N = 200(%)	
1 - Nulliparous, single cephalic, - > 37 weeks, in spontaneous labor	34 (17)	17 (8.3)	3 (1.5)	<0.001F
2 - Nulliparous, single cephalic, - > 37 weeks, induced or C-Section	17 (8.5)	17 (8.3)	14 (7)	
3 - Multiparous (excluding previous C-Section), single cephalic, - > 37 weeks, in spontaneous labor	23 (11.5)	5 (2.4)	4 (2)	
4 - Multiparous (excluding previous C-Section), single cephalic, - > 37 weeks, induced or C-Section before labor	29 (14.5)	8 (3.9)	17 (8.5)	
5 - Previous C-Section, single cephalic, - > 37 weeks	37 (18.5)	84 (41)	96 (48)	
6 - All nulliparous breeches	12 (6)	8 (3.8)	1 (0.5)	
7 - All multiparous breeches (including previous C-Section)	12 (6)	8 (3.9)	4 (2)	
8 - All multiple pregnancies (including previous C-Section)	7 (3.5)	4 (2)	1 (0.5)	
9 - All abnormal lies (including previous C-Section)	5 (2.5)	2 (1)	1 (0.5)	
10 - All single cephalic, - < 37 weeks (including previous C-Section)	24 (12)	52 (25.4)	59 (29.5)	

^F^Fisher exact test for association.

## Results of the qualitative component of the study

In all, 19 IDIs were conducted, out of which 13 were conducted with women who had undergone C-Section (second postoperative day), three with HCPs performing C-Sections, and three with management representatives of the participating HCSs. The following main themes emerged from the qualitative interviews:

### Preference for the mode of birthing and birth preparedness

A majority of the participants, particularly multiparous women with previous experience of C-Section and vaginal birth indicated their preference for vaginal birth. Various reasons supported their choice of vaginal birth, the most frequent reason being a prolonged recovery times after C-Section. One of the participants commented:

*“…their (women’s) health gets affected, they might not be as active (after C-Section), they go through the pain of cesar (C-Section), then they have (other) children to look after…”.*

Another respondent who experienced both vaginal birth and C-Section mentioned:

*“…woman is up and about 2 hours after normal (birth), but recovery after operation(C-Section) may take 3 months….”.*

C-Section was more of a challenge for working mothers since the prolonged recovery time limits their ability to rejoin their work, which may also have financial consequences. According to one of the respondents who works outside the home:

“*…the only concern is how long the recovery period will be…because being a working woman, reverting back to the previous routine is a concern…”*

However, most of the participants believed that once a woman undergoes C-Section, she will end up having C-Section for all her subsequent pregnancies. According to a respondent:

*“…I had one operation (C-Section) so they (private HCP) said that now every time I will have to undergo a Caesarean section…”*

Labour pain during vaginal birth was one of the main reasons women opted for C-Section. One of the respondents opined:

*“…Girls get anxious by the process of natural birth, therefore they opt for C-Section. They can’t bear the pains of normal birth…”*

Another respondent who had given birth at the private HCS shared her own experience:

*“… I felt that the operation is better in terms of pain. I was a bit scared of the pains of natural birth and that I might not be able to tolerate this pain …”*

When enquired about what can be done to reduce this fear that may compel women to opt for C-Sections, the same respondent replied:

*“… women should be informed that during natural birth, you will have pain for this much time, but then they should also be told about the benefits…natural process by Allah (God) is the natural vaginal birth…doctors should use pain-reducing methods…”*

One of the HCPs from the semi-private facility opined that private HCSs are in a better position to ensure counselling during the perinatal period, which may be beneficial in reducing unnecessary C-Sections:

*“…I think this (counselling) might not be possible in tertiary, government, or semi-private setups because they are already too over-burdened; however, the private sector can initiate this…”*

One of the representatives of the management of the public HCS acknowledged their lack of resources to address the issue of counselling during the antenatal period:

*“…We have limited manpower...we need some counsellors… especially in our clinics, in our antenatal wards…if they (counsellors) just give them (women)5 minutes, repeatedly counsel them, it will definitely make a difference…”*

### Sub-optimal contraception and C-Section

Women commented on their unmet needs for contraception. Women who have had multiple previous C-Sections were left with no choice but to undergo another C-Section, as a trial of induction of labor was not medically recommended for them. One of the respondents shared her experience:

*“…I suddenly found about my pregnancy… I took medicines to abort but nothing happened…I took a lot of medicines, herbs, and even drank kahwa (a special tea used traditionally for abortion)…”*

One of the respondents reported undergoing C-Section 13 months after her last C-Section as she refused to opt for family planning methods. She opined:

*“…One should not use these methods (family planning) otherwise problems begin in one’s body. One should be careful (not to conceive)…”*

The HCPs also addressed this issue of sub-optimal utilization of contraceptive services and risks of repeated C-Sections. One of the HCPs from the public-sector hospital expressed her concerns:

*“…imagine how hard it would be for a woman who just had a C-Section and she is coming back after puerperium with a positive pregnancy test and her next child will be born after 10 months… ”*

### Another HCP belonging to the private sector mentioned

*“…repeated C-Section will affect their (women’s) health the most. The main issue will be their health and then all their household, their routine, everything will be disturbed…”*

### Mode of birthing preferred by different healthcare sectors (Public, Private and Semi-*private)*

Women had different opinions when it came to their experience of giving birth in different healthcare sectors. One of the women who gave birth at the private HCS shared her experience:

*“…They (private HCPs) said that it was an operation case, so they did it. They didn’t get into the hassle of attempting vaginal birth, they prefer to operate …”*

### Another respondent stated

*“…most of them (private HCPs) prefer C-Section. I don’t know about the benefit (laughs) I think they make their money, obviously C-Section has more cost, and more so if the case is on company penal.”*

### A respondent who delivered at the semi-private HCS commented

“*Especially in Pakistan, they have made it a business (laughs). Now it’s everywhere. They (doctors) immediately go for C-Section.”*

The opinions and experiences of the participants were different about the birthing mode at the public HCS. A respondent who had a C-Section at the public HCS shared her experience:

*“…they (government hospitals) always try to make efforts for a normal case (birth). Even now when I got my name registered, everyone was saying that your fourth baby would be delivered normally (laughs), but I said no…”*

However, women who gave birth at the private HCS expressed their satisfaction with the antenatal care and opined that private HCSs may be better equipped with resources to facilitate women through counselling to improve their well-being. According to one of the respondents:

*“…my doctor gave me instructions on how to sit or lie and what to eat and what not to eat when I used to go for my appointments…my doctor guided me about everything…”*

One of the representatives of the public HCS commented on the lack of regulation and audit of C-Sections, especially in private HCSs:

*“…in our setting (public hospital) whenever there is a C-Section, we analyse to understand the reasons why this section was conducted…since we have limited resources, we can’t afford either at the financial or human resource level, nor in terms of time… but they (private HCS) have no regulation… we will have to regulate them and penalize them (for performing unnecessary C-Sections)….”*

## Discussion

This study examined the patterns of C-Section deliveries and stakeholder-perceived drivers of current C-Section practices against the WHO 10-group classification system, and explored the perceptions of stakeholders towards the current C-Section practices in public, private and semi-private HCSs in Karachi, Pakistan. The findings of the quantitative component of this study reveal that a prior history of C-Section was the most common reason for subsequent C-Sections in all three HCSs, while the qualitative component of this study reveals that a majority of women do not prefer C-Section mainly due to the prolonged recovery time associated with it; however, pain associated with vaginal birth may be a compelling factor for women to opt for C-Section.

In this study, Group 5 of the WHO 10-Group classification system was found to be the greatest contributor in all HCSs, followed by Group 10 in public and semi-private, and Group 1 in private HCSs. These results are supported by findings of other global and regional studies [20,21]. A full-term, cephalic pregnancy with a previous C-Section (Group 5) highlights the need to implement clinical and non-clinical interventions to reduce the C-Section rate in this group. An interesting finding that emerged from the qualitative component of this study was women’s understanding that once a woman undergoes C-Section, all her prospective deliveries will be through C-Section. However, studies have reported that a trial of labour after Caesarean (TOLAC) can lead to a successful vaginal birth C-Section (VBAC), with efforts directed at implementing strategies to prevent the first C-Section in Groups 1–4 [21,22]. The IDIs highlighted that the pain associated with vaginal birth could compel women to opt for an elective C-Section; therefore, there is a need for counselling for birth preparedness and pain management techniques to reduce the risks of elective C-Sections.

According to Robson et al., the contribution of Group 10 should ideally be 4–5% of the cases. Higher proportions suggest a tertiary referral centre or a potentially high risk of premature birth [23,24]. This was evident by the results reported in this study, where Group 10 was the second most common contributor in the public and semi-private HCS, which receive referred and complicated cases from secondary HCSs. Group 1, which includes full-term nulliparae with a single cephalic fetus and spontaneous labour, was the second most common group contributing to the overall C-Section rate in the selected private HCS. Financial incentives associated with performing C-Sections may be a factor for some private HCPs to perform C-Sections. The data from Pakistan Demographic and Health Surveys (1990–2018) showed that women from higher socio-economic groups have the financial resources to afford costly private healthcare and the expenditures of elective C-Sections [25].

This study also found that a majority of the women who delivered at the public HCS had an unplanned pregnancy. Literature suggests that there may be a significant relationship between unintended pregnancy and C-Sections [26]. According to the guidelines by the American College of Obstetrics and Gynaecologists, women with a history of C-Sections, especially those who are appropriate candidates for TOLAC should be counselled that a shorter inter-pregnancy interval might increase their risk of uterine rupture and other complications, increasing their chances of another C-Section [27]. Our study also found that antenatal counselling on planned pregnancy could be a modifiable factor and an effective non-clinical intervention in reducing the rates of unnecessary C-Section in our local context.

In 2018, the WHO published its evidence-based recommendations on non-clinical interventions to reduce unnecessary C-Sections [15]. The findings of this current study provide evidence about the relevance and need to implement the WHO-recommended non-clinical interventions to reduce unnecessary C-Sections in Pakistan. In line with the WHO recommendation to improve the education of pregnant women and the community, the current study also reports the need expressed by the study participants to provide educational and counselling services for birth preparedness and labour pain management. Moreover, in the current study, there was an expressed need for better regulation and audit of C-Sections to continuously monitor the actual need for performing C-Sections, especially in the private HCS, and to consider family planning as an effective tool in increasing inter-birth spacing and thus reducing the chances of potential C-Sections.

To the best of our knowledge, this study was the first to assess the effectiveness of the WHO 10-Group classification system to identify patterns of C-Sections practices in public, semi-private and private HCSs in Karachi, Pakistan, and to qualitatively explore stakeholders’ perceived drivers of these patterns and practices in Pakistan. We recognise a few limitations of this study. The foremost was our inability to estimate the relative size of each Robson group or to compare women who underwent C-Sections with women who had vaginal birth. This limited our ability to report the actual number of unnecessary cases of C-sections. Since this study was conducted at a single public tertiary health facility with a significant burden of referred cases, some of the findings might not be generalizable. This justifies the need for future research in other large public and private HCSs across Pakistan.

## Conclusions

This study identified patterns of C-Sections and perceptions of stakeholders regarding the drivers of current C-Sections practices in Pakistan. This study also generated evidence on the utilization of the WHO-recommended, evidence-based interventions for reducing C-Sections in Pakistan. Group 5 of the WHO 10-Group classification system was found to be the greatest contributor to the overall C-Section cases in all HCSs, followed by Group 10 in public and semi-private, and Group 1 in private HCSs. Stakeholders expressed need to implement WHO-recommended guidelines on non-clinical interventions to reduce C-Sections in Pakistan.

## Supporting information

S1 File Data tools: Data collection forms. (DOCX)

S1 Data Data. (XLSX)

S1 Text Codes. (DOCX)
