# Supplementary material for: Patterns and stakeholder-perceived drivers of Caesarean section practices: Evidence from a multicenter study in a lower-middle-income country
Source: PLOS Glob Public Health. 2026 May 14;6(5):e0006274. doi: 10.1371/journal.pgph.0006274 (PMC13175372; doi:10.1371/journal.pgph.0006274)
Supplement: S1 File — (DOCX) [file pgph.0006274.s001.docx]

**Data collection forms**

**The WHO 10 group classification system to reduce unnecessary Cesarean Sections: evidence from a multicenter study in Karachi, Pakistan**

**PART A: Socio-economic Demographics**

1. Please provide your age: _____________years
2. What is your marital status:

- Married
- Divorced
- Separated
- Widowed

1. What religion do you practice:

- Islam
- Christianity
- Hinduism
- Sikhism
- Other _____________
- None

1. Years of full time education

- None
- Primary
- Secondary
- Graduate
- Post graduate

1. What is your current employment status:

- Employed/self employed
- Unemployed

1. What is your total household income: PKR _____________________

1. Was this pregnancy planned?

- Yes
- No

1. How many children do you have, including the newborn

- You currently have ____ girl/s
- You currently have ____boy/s

**PART B: Pregnancy- related information**

1. Have you ever had an abortion :

- Yes
- No

1. Have you ever had a C-section :

- Yes
- No

1. Considering the recent pregnancy, did you have a preference for any particular sex:

- Yes, I wished to have a daughter
- Yes, I wished to have a son
- No, I had no particular preference

1. Did you have any particular concerns about your recent pregnancy?

- Yes
- No

1. Did you have any complications during your recent pregnancy?

- Yes
- No

1. If yes, the type of complication that you had during your recent pregnancy:

- Hypertensive Disorders of Pregnancy
- Gestational diabetes
- Infections
- Anemia
- Threatened abortion
- Amniotic fluid complications
- Bleeding
- Ectopic pregnancy
- Placental complications
- Any other, please elaborate_______________________

1. Number of antenatal care visits during your recent pregnancy

- None
- 1-3
- 4 or more

1. Place of delivery

- Public
- Private

**PART C: delivery - related information**

1. Gestational age of the foetus at birth

- < 37 weeks
- ≥ 37 weeks
- Do not know

1. Gestational age of the foetus at birth

- < 37 weeks
- ≥ 37 weeks
- Do not know

1. Onset of labour

- Spontaneous
- Induced
- C- section before onset of labor

1. Foetal presentation/lie

- Cephalic
- Breech
- Transverse/Oblique

1. Number of foetus

- Single
- Multiple

1. Mode of C-section

- Emergency
- Scheduled

1. Foetal outcome

- Live birth
- Still birth

1. Birth weight

- <1000 grams
- 1,000–2,499
- 2,500-3,999
- ≥ 4,000

1. Reason for performing the last C-section

- Previous C- Section
- Foetal Distress
- Hypertensive Disorders of Pregnancy
- Failed Induction of Labour
- Cephalo-pelvic Disproportion
- Maternal Requests
- Contracted Pelvis
- Breech
- Abruption
- Placenta Previa
- Others, please specify_____________

**Pre-operation clinical notes**

______________________________________________________________________________________________________________________________________________________________________________________________________________________________________________________________________________________________________________________________________________________________________________________________________

**Post-operation clinical notes**

____________________________________________________________________________________________________________________________________________________________________________________________________________________________________________________________________________________________________________________________________________________________________________________________________________________________________________________________________________________

**The WHO 10 group classification system to reduce unnecessary Cesarean Sections: evidence from a multicenter study in Karachi, Pakistan**

**In-depth interview guidelines**

- Introduction of the interviewer and the study.
- Obtaining verbal consent before recording the interviews and reassurance of confidentiality.
- Assuring the participants that they can stop the interview at any time.
- Keeping the interactions focused and at the same time allowing participants’ perceptions and experiences to emerge.
- Thanking the participants for their time and willingness to share their experience at the end of the interview.
- If requested by the participants, provide more information about the topic of discussion and guidance on where to seek additional help.

**Interview categories for postnatal women**

- Personal experience of the latest pregnancy (planned/unplanned, issues and challenges during the last pregnancy, overall health and well-being during the recent pregnancy, any complications, if so coping up strategies, any socio-economic issues of concern during the recent pregnancy)
- Personal experience of the latest C-section (planned/unplanned, issues and challenges leading to C-section, overall experience, any complications, decision to opt for C-sections, partner’s involvement in decision making and support, recovery)
- Socio-economic costs associated with C-sections, any relevant concerns, Social emotional support provision/barriers, etc. during antenatal and postnatal period
- Perceptions and experiences of healthcare providers and healthcare facility management behaviour during the last C-section and post-operation period
- Perceptions about the ‘Need of C-section’ during the last delivery
- Thoughts, perceptions and suggestions about non-clinical interventions to avoid unnecessary C-sections and their adaptability in Pakistan
- Child-related issues (overall health of the new born, coping with new routine post-operation, breast-feeding practices, etc…)

**Interview categories for healthcare providers performing C-sections**

- Issues and challenges pertaining to choice of delivery at the professional, institutional and local context
- Perceptions about current situation of rates and practices of C-section in Pakistan
- Perceptions about pregnant women’s choice of delivery (reasons, challenges, opportunities) and institutional preference for a particular type of delivery
- Any thoughts on reduction of C-sections and opinions to reduce unnecessary C-sections, based on professional experience and practice
- Perceptions and suggestions about global evidence on non-clinical interventions to avoid unnecessary C-sections and their adaptability in Pakistan

**Interview categories for healthcare facilities providing C-section services**

- Issues and challenges pertaining to choice of delivery at the institutional and local context
- Perceptions about current situation of rates and practices of C-section in Pakistan
- Perceptions about pregnant women’s choice of delivery (reasons, challenges, opportunities) and healthcare provider’s behaviours and practices for a particular type of delivery
- Any thoughts on reduction of C-sections and opinions to reduce unnecessary C-sections, based on professional experience and practice
- Perceptions and suggestions about global evidence on non-clinical interventions to avoid unnecessary C-sections and their adaptability in Pakistan
