# Supplementary material for: Patterns and stakeholder-perceived drivers of Caesarean section practices: Evidence from a multicenter study in a lower-middle-income country
Source: PLOS Glob Public Health. 2026 May 14;6(5):e0006274. doi: 10.1371/journal.pgph.0006274 (PMC13175372; doi:10.1371/journal.pgph.0006274)
Supplement: S1 Text — (DOCX) [file pgph.0006274.s003.docx]

| S.No | Code | value |
| --- | --- | --- |
| 1 | A1 | Age |
| 2 | A2 | Marital status |
| 3 | A3 | Religion |
| 4 | A4 | Education |
| 5 | A5 | Employment |
| 6 | A6 | Income |
| 7 | A7 | Have children |
| 8 | A8 | #of girls/ #of boys |
| 9 | A9 | Total #of children alive |
| 10 | B1 | History of abortion |
| 11 | B2 | History of C-section |
| 13 | B3 | Child gender preference |
| 14 | B4 | Planned pregnancy |
| 15 | B5 | Antenatal complications |
| 16 | B6 | Had concerns about recent pregnancy |
| 17 | B7 | Antenatal care visits |
| 18 | B8 | Place of delivery |
| 19 | C1 | Onset of labour |
| 20 | C2 | Presentation |
| 21 | C3 | #of fetus |
| 22 | C4 | Mode of CS |
| 23 | C5 | Fetal outcome |
| 24 | C6 | Birth weight |
| 25 | C7 | Reason for CS |
| 26 | C8 | Complications during delivery |
| 27 | C9 | Robson’s classification |
